# Ursolic acid suppresses triple-negative breast cancer progression through mediating FABP4/PPARG pathway

**DOI:** 10.1186/s40001-025-02794-y

**Published:** 2025-07-02

**Authors:** Ying Deng, Junyi Wu, Di Pan, Wan Su, Songpo Wang

**Affiliations:** 1 Department of Traditional Chinese Medicine, Shanghai General Hospital, Shanghai, 200080 China; 2 Department of General Surgery, Shanghai General Hospital, Shanghai, 200080 China; 3 Department of Oncology, Longhua Hospital Shanghai University of Traditional Chinese Medicine, Shanghai, 200032 China

**Keywords:** Ursolic acid, Triple-negative breast cancer, FABP4/PPARG pathway, Proliferation, Migration

## Abstract

**Background:**

Triple-negative breast cancer (TNBC) remains the deadliest subtype of breast cancer owing to high metastatic potential and poor prognosis. Herein, we examined the antitumor effects of ursolic acid (UA), a pentacyclic triterpene compound, against TNBC and the underlying mechanisms.

**Methods:**

TNBC cells were exposed to a graded concentration of UA, and cell proliferation and migration were examined through CCK-8 and wound healing assays. Transcriptome data of 116 TNBC and 290 normal tissues were acquired for determining differentially expressed genes. Using the PubChem and the SwissTargetPrediction, potential UA targets were inferred. 10 pairs of human TNBC and normal tissues were gathered for examining the expression of UA targets FABP4 and PPARG. The influence of FABP4/PPARG knockdown and overexpression on the therapeutic effects of UA was then observed.

**Results:**

UA treatment hampered proliferation and migration of TNBC cells in a concentration-based fashion. FABP4 and PPARG were determined as targets of UA. Their expression levels were gradually elevated as the increase of UA concentration. Clinically, TNBC tumor tissues displayed notable down-regulation of FABP4 and PPARG in comparison with normal tissues. UA treatment increased PPARG expression and promoted its activation, which could be effectively attenuated by FABP4 knockdown. In addition, the efficacy of UA on suppressing TNBC cell growth and migration was notably reversed and enhanced by FABP4/PPARG knockdown and overexpression, respectively.

**Conclusions:**

This study suggests that UA treatment increases PPARG expression through modulating FABP4, thus preventing TNBC progression, expanding the clinical application of UA and providing a theoretical basis for its usage in TNBC treatment.

**Supplementary Information:**

The online version contains supplementary material available at 10.1186/s40001-025-02794-y.

## Introduction

Triple-negative breast cancer (TNBC) occupies 10–15% of all breast cancer patients and remains the deadliest subtype of breast cancer owing to high metastatic potential and poor overall survival [1, 2]. Because of the deficiency of specific molecular targets, TNBC does not respond to endocrine therapy or anti-HER2 therapy [3]. Novel therapeutic strategies of TNBC, comprising targeted therapy, such as CDK4/6 inhibitors and PARP inhibitors as well as immunotherapy, are being tested in current clinical trials. Although chemotherapy is a primary systemic treatment, its outcomes are often unsatisfactory because of high recurrence risk and undesirable prognostic outcomes [4, 5]. Hence, it’s urgently required innovative treatment strategies and drugs to treat TNBC.

Fatty acid binding proteins (FABPs), a class of cytoplasmic proteins, enable to promote the transport and response of lipids, with crucial functions in fatty acid uptake and directing lipids to specific cellular components. Distinct FABP family members display unique tissue expression patterns, mainly expressed in tissues actively involved in lipid metabolism [6]. Among them, FABP4 was first discovered in mature adipocytes and adipose tissues [7]. Accumulated evidence demonstrates that FABP4 as a novel player in breast cancer [8–11]. Peroxisome proliferator-activated receptor gamma (PPARG) acts as a crucial factor in modulating lipid metabolism, energy homeostasis, and immune responses [12]. There is a positive feedback loop between FABP4 and PPARG in facilitating fatty acid uptake and oxidation in cholangiocarcinoma lymph node metastasis [13]. PPARG has been determined as a promising biomarker of breast cancer following validation of its down-regulation in breast cancer and its connections to survival outcomes [14]. Experimental evidence demonstrates that overexpression of PPARG can attenuate TNBC progression [15], uncovering the potential of PPARG as a therapeutic target.

Ursolic acid (UA), a pentacyclic triterpene compound, is extensively found in medicinal plants, fruits, herbs and other plant sources, characterized by unique pentacyclic structure and hydrophobic tail [16]. The lipophilicity of UA leads to its oral bioavailability, and UA is primarily absorbed in the gastrointestinal tract and disseminated throughout the body [17]. UA possesses multiple pharmacological characteristics, e.g., anti-proliferation, anti-inflammation, anti-oxidation, anti-metastasis and anti-resistance, making it an effective treatment agent against breast cancer [18–24]. Owing to its broad-spectrum biological activities, UA holds an important position among numerous triterpenoids. Nevertheless, its mechanism of action remains largely unclear in the treatment of TNBC. On the basis of this background, this study assessed the antitumor efficacy of UA against TNBC as well as the underlying mechanisms, and reported that UA suppressed TNBC progression through mediating FABP4/PPARG pathway.

## Materials and methods

### Cell culture and compounds

Human TNBC cell lines MDA-MB-231 (HCL-0059, CTCC, Hangzhou, China) and MDA-MB-468 (HCL-0060, CTCC) as well as human normal mammary epithelial cell line MCF-10A (CTCC-001–0045, CTCC) were cultivated in DMEM medium (SH30243.01, Hyclone, South Logan, UT, USA) plus 10% FBS (A511-001, Lonsera, Shanghai, China) and 1% penicillin–streptomycin solution (BL505A, Biosharp, Hefei, China) in a 5% CO_2_ incubator at 37 °C. UA (B21403, Yuanye, Shanghai, China) was dissolved by DSMO. DMSO was used as a vehicle control.

### Cell proliferation assay

Cells were inoculated into a 96-well plate (5 × 10^3^ cells/well) and grown for 24 h. Next, 0, 5, 10, 20, and 30 μM UA was added and cultured for 24, 48, and 72 h. Each well was added by 20 μL of Cell Counting Kit-8 (M006, CTCC), followed by incubation for 1 h away from light. OD value was examined at 450 nm utilizing MK3 microplate reader (Thermo Fisher, Waltham, MA, USA).

### Wound healing assay

Cells were inoculated into a six-well plate and grown to confluence. They were scratched utilizing a sterile 200 μl pipette tip to make a wound. The scratched cells were removed, and serum-free medium was added. The wound healing process was photographed at 0 and 48 h.

### Bioinformatics analysis

#### Data acquisition

RNA transcriptome data of TNBC were from the TCGA portal (https://tcga-data.nci.nih.gov/tcga/). Totally, 116 TNBC and 290 normal tissue specimens were included in the present study.

#### Differential expression analysis

With limma package [25–27], differentially expressed genes (DEGs) in TNBC versus normal tissues were analyzed, with adjusted *p* value < 0.05 and |log2 fold-change|> 1 as the screening criteria.

#### Functional enrichment analysis

Gene Ontology (GO) and Kyoto Encyclopedia of Genes and Genomes (KEGG) enrichment analyses of DEGs were executed utilizing clusterProfiler package [28].

#### UA target prediction

The chemical structure of UA was obtained from the PubChem (https://pubchem.ncbi.nlm.nih.gov) [29]. Utilizing the chemical structure, potential targets of UA were predicted by use of the SwissTargetPrediction (http://www.swisstargetprediction.ch) [30]. Through intersecting with DEGs, UA targets were finally determined, and UA-target network was visualized via Cytoscape [31].

#### Protein–protein interaction analysis

UA targets were analyzed by the STRING (https://string-db.org/) [32]. By default, protein–protein interactions of UA targets were acquired, with establishment of a protein–protein interaction network.

#### qRT-PCR

Total RNA was extracted utilizing Trizol extraction kit (15596018, Invitrogen, Carlsbad, CA, USA), with subsequent RNA concentration detection. cDNA was synthesized using 5 μl total RNA, 1 μl random primer, 5 μl Rnase-free ddH_2_O, 4 μl 5 × reaction buffer, 2 μl dNTP Mix (10 mmol/L), 1 μl Rnase inhibitor (20 U/μl), and 2 μl reverse transcriptase (10 U/μl). Primer sequences included: human *FABP4*, 5’-AGTCAAGAGCACCATAACC-3’ (forward), 5’-TTCCACCACCAGTTTATCAT-3’ (reverse); human *PPARG*, 5’-CGAAGACATTCCATTCACAA-3’ (forward), 5’-CCACAGACACGACATTCA-3’ (reverse); human *GAPDH*, 5’-GGAGCGAGATCCCTCCAAAAT-3’ (forward), 5’-GGCTGTTGTCATACTTCTCATGG-3’ (reverse). qRT-PCR was conducted using 7 μl ddH_2_O, 10 μl 2 × SYBR Green qPCR Master Mix, 1 μl forward primer (10 μM), 1 μl reverse primer (10 μM), and 1 μl cDNA. The result was analyzed with real-time detector (ABI 7500, Beijing, China). Relative expression of *FABP4* and *PPARG* was estimated with 2^−△△CT^ method, with *GAPDH* as a control.

#### Western blot

Cell extracts were acquired utilizing RIPA lysis (P0013B, Beyotime, Shanghai, China) plus PMSF (BP2655, RUIBIO, Beijing, China), followed by protein quantification via BCA method (BL521A, Biosharp, Beijing, China). The extracted protein was separated and then transferred onto PVDF membrane (IPVH00010, Millipore, Billerica, MA, USA). Following blockade, the membrane was incubated with primary antibody against FABP4 (1:5000, 12802–1-AP, Proteintech, Wuhan, China), PPARG (1:1000, 16643–1-AP, Proteintech), and GAPDH (1:5000, 60004–1-Ig, Proteintech) overnight at 4 °C and subsequently incubated with HRP-labeled goat anti-rabbit and anti-mouse IgG (H + L) (1:5000, ZB-2301 and ZB-2305, Zhongshan Jinqiao, Beijing, China) for 1 h at 37 °C. ECL solution (ECL-03–250, 7sea biotech, Shanghai, China) was added to the membrane. Images were acquired with integrated chemiluminescence imager (ChemiScope 5300 Pro, CLINX, Shanghai, China).

#### Patient samples

Totally, 10 pairs of TNBC tumor specimens and adjacent normal tissues were collected from Shanghai General Hospital. All patients were histopathologically diagnosed as TNBC. Following the Declaration of Helsinki, this study acquired the approval of the Ethics Committee of Shanghai General Hospital (K-2024–323), with each patient providing written informed consent.

#### Immunohistochemistry

Paraffin-embedded tissue sections were deparaffinized at 60 °C for 20 min and cleared in xylene, followed by rehydration by a series of alcohol series. To block endogenous peroxidase, 3% hydrogen peroxide drops were added to the sections, with subsequent incubation at room temperature for 15 min. Following blockade utilizing normal goat serum at room temperature for 30 min, the sections were incubated with primary antibody against FABP4 (1:2000, 12802–1-AP, Proteintech), and PPARG (1:1000, 81490–5-RR, Proteintech) overnight at 4 °C as well as incubated with HRP-labeled goat anti-rabbit and anti-mouse IgG (H + L) (1:5000, ZB-2301 and ZB-2305, Zhongshan Jinqiao) for 30 min at 37 °C. DAB solution (ZLI-9018, Zhongshan Jinqiao) was added to the sections. After hematoxylin staining and dehydration, the sealing was carried out. Images were viewed or collected under a microscope (XDS-1A, CANY, Shanghai, China).

#### Cell transfection and treatment

Cells were inoculated into a 6-well plate (5 × 10^5^ cells/well). After the cell density was ~ 80%, 2 ml fresh culture medium was added to each well. 125 μl serum-free culture medium, 100 pmol siRNA/scramble siRNA control (si-NC), and 4 μl Lipo8000™ transfection reagent (C0533, Beyotime) were mixed, with 125 μl mixture adding to each well. Then, the cells were exposed to 30 μM UA for 48 h. The sequences were as follows: si-FABP4#1, 5’-GCAGCAUCAAGAAGAAUCATT-3’ (sense), 5’-UGAUUCUUCUUGAUGCUGCTT-3’ (antisense); si-FABP4#2, 5’-CUGAUCUACUACUACUACTT-3’ (sense), 5’-GUAGUAGUAGUAGAUCAGTT-3’ (antisense); si-FABP4#3, 5’-GAAGUCUGUGAAGAACUGATT-3’ (sense), 5’-UCAGUUCUUCACAGACUUCTT-3’ (antisense); si-NC, 5’-UUCUCCGAACGUGUCACGUTT-3’ (sense), 5’-ACGUGACACGUUCGGAGAATT-3’ (antisense).

To overexpress FABP4 and PPARG, the coding sequence sequences of FABP4 and PPARG were cloned into the pLenti-CMV-puro plasmid (Invitrogen, USA), with empty vector as a control. After transfection for 24 h, the cells were exposed to 30 μM UA for 48 h.

#### Immunofluorescence

Cells were grown on glass coverslips, with subsequent 15-min fixation by 4% paraformaldehyde (P0099, Beyotime). Afterwards, they were permeabilized by 0.1% Triton (P0096, Beyotime) for 10 min. Following blockade by 10% normal goat serum at 37 °C for 1 h, they were incubated with primary antibody against PPARG (1:50, 81490–5-RR, Proteintech) overnight at 4 °C, which were detected by FITC-conjugated goat anti-rabbit IgG (H + L) (1:100, SA00003-2, Proteintech). Nuclear staining was conducted with DAPI (C1002, Beyotime) for 15 min at room temperature. Microscopic analyses were conducted utilizing an immunofluorescence microscope (IX73, OLYMPUS).

#### Statistical analysis

All experiments were carried out independently at least three times. Bioinformatics analysis was implemented through R software (version 4.2.0), while statistical analysis of experiments was performed with GraphPad Prism (version 9.0.0). One-way analysis of variance with Tukey post-test was adopted for multiple comparisons, with unpaired *t* test for comparing two groups. Differences were considered significant when *p* ≤ 0.05.

## Results

### UA treatment attenuates TNBC cell proliferation and migration in a concentration-based fashion

**Fig. 1:**
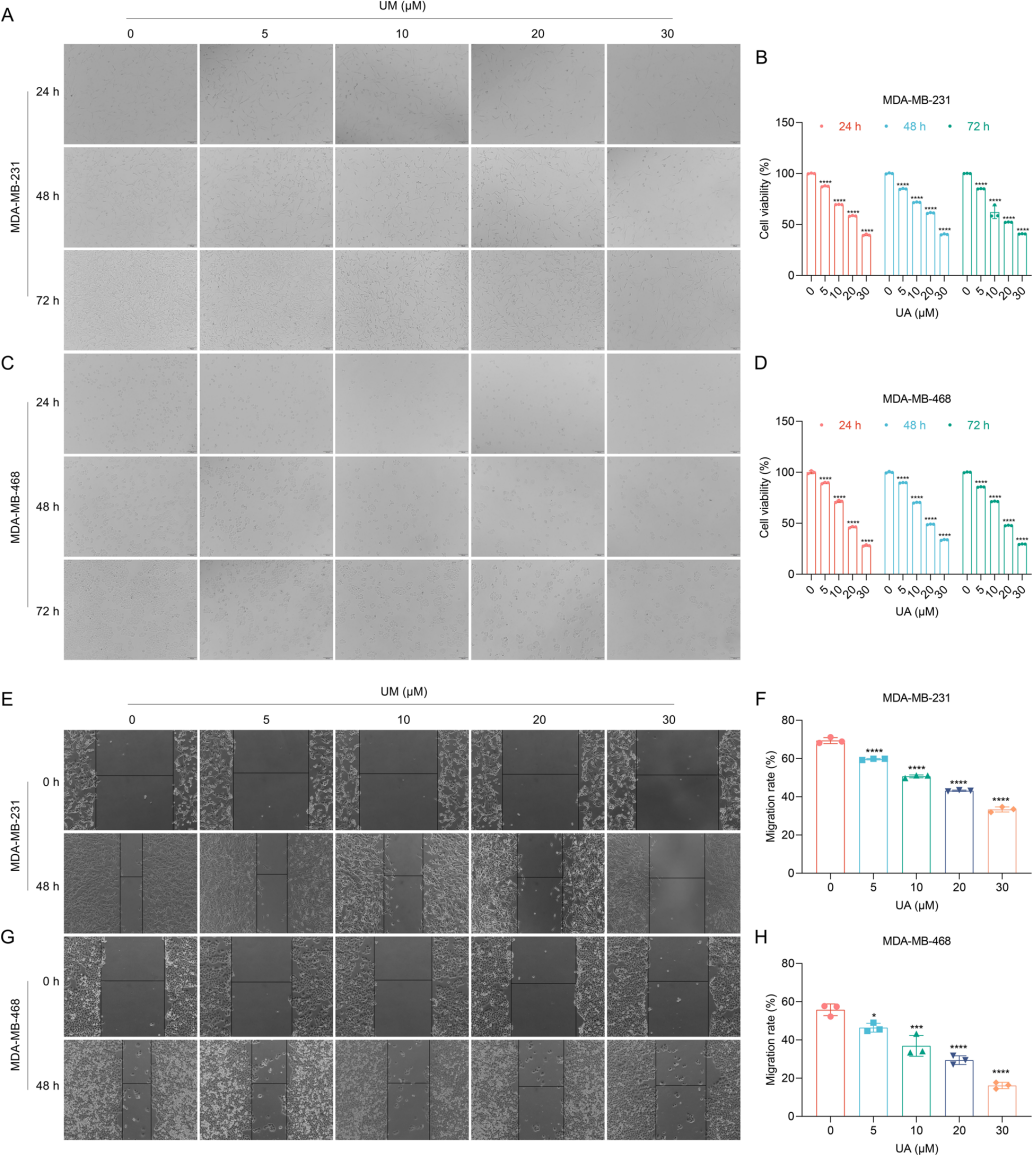
UA administration attenuates proliferation and migration of TNBC cells in a concentration-dependent fashion. **A** Photographs of MDA-MB-231 cells with exposure to 0, 5, 10, 20, and 30 μM UA that was dissolved by DMSO for 24, 48, and 72 h. DMSO was used as a vehicle control. Scale bar, 100 μm. **B** Cell proliferation of MDA-MB-231 cells with exposure to 0, 5, 10, 20, and 30 μM UA for 24, 48, and 72 h. **C** Photographs of MDA-MB-468 cells administrated with 0, 5, 10, 20, and 30 μM UA for 24, 48, and 72 h. Scale bar, 100 μm. **D** Cell proliferation of MDA-MB-468 cells administrated with 0, 5, 10, 20, and 30 μM UA for 24, 48, and 72 h. **E** Photographs of MDA-MB-231 cells with exposure to 0, 5, 10, 20, and 30 μM UA after scratching at 0 h, and 48 h. Scale bar, 100 μm. **F** Calculation of migration rate of MDA-MB-231 cells with exposure to 0, 5, 10, 20, and 30 μM UA for 48 h. **G** Photographs of MDA-MB-468 cells administrated with 0, 5, 10, 20, and 30 μM UA after scratching at 0 h, and 48 h. Scale bar, 100 μm. **H** Calculation of migration rate of MDA-MB-468 cells administrated with 0, 5, 10, 20, and 30 μM UA for 48 h. *n* = 3 per group. *, *p* < 0.05; ***, *p* < 0.001; ****, *p* < 0.0001 from one-way analysis of variance with Tukey post-test

To uncover the antitumor property of UA against TNBC, this study administrated MDA-MB-231 and MDA-MB-468 TNBC cells with a graded concentration of UA (0, 5, 10, 20, and 30 μM) for distinct timepoints (24, 48, and 72 h). Consequently, TNBC cell proliferation was notably attenuated by UA in a concentration-based fashion (Fig. 1A–D). However, our data suggested the time independent effect as there was minimal variation in response between 24, 48 and 72 h. In line with a previous study [19], we selected 48 h for subsequent analysis. Wound healing assay showed that 0-h and 48-h scratches of TNBC cells exposed to a graded concentration of UA (0, 5, 10, 20, and 30 μM) were observed, and UA remarkably weakened TNBC cell migration in a concentration-based manner (Fig. 1E–H). To validate the TNBC specificity, normal mammary epithelial cells (MCF-10A) were included for CCK-8 after treatment with a graded concentration of UA for distinct timepoints. It was proven that UA treatment did not significantly impact the proliferation of normal mammary epithelial cells (Supplementary Fig. 1A, B). Altogether, UA enables to attenuate growth and migratory potential of TNBC cells in a concentration-based fashion.

### Identifying potential UA targets in the treatment of TNBC

**Fig. 2:**
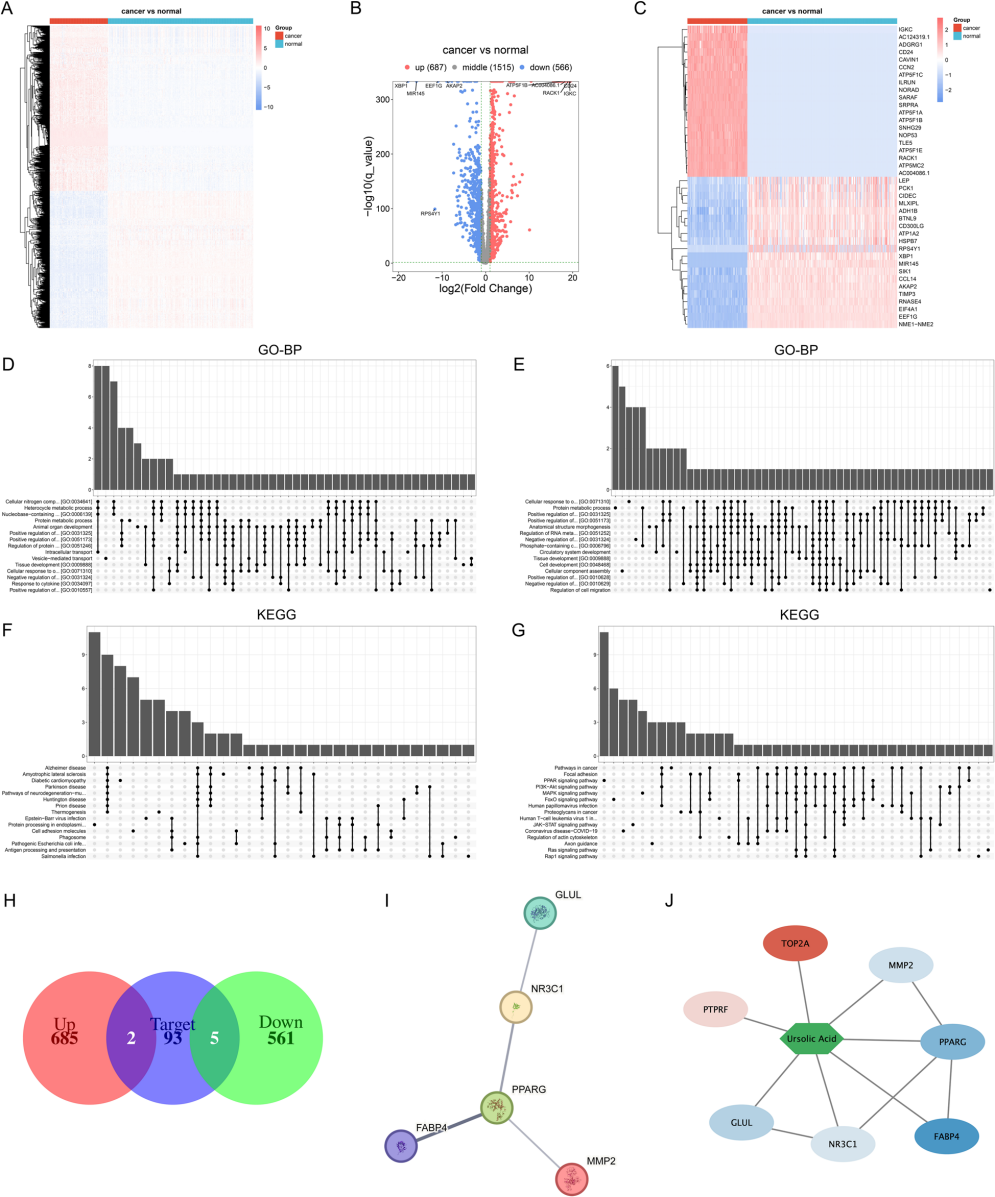
Identifying potential UA targets in the treatment of TNBC. **A, B** Heatmap and volcano plots depicting DEGs between TNBC and normal tissues. Red, up-regulation; blue, down-regulation. **C** Heatmap of the top 20 up- and down-regulated DEGs in TNBC versus normal tissues. **D**, **E** GO biological processes (GO-BP) enriched by up- and down-regulated DEGs. **F**, **G** KEGG pathways enriched by up- and down-regulated DEGs. **H** Venn diagram of the DEGs and SwissTargetPrediction-predicted UA targets. **I** Protein–protein interaction network of UA targets. **J** UA-target network. The green hexagon represents UA, and the oval nodes represent UA targets. Red and blue represent up- and down-regulated DEGs in TNBC, respectively. The color shade represents the differential expression from high to low

We gathered RNA transcriptome data of 116 TNBC and 290 normal tissues from the TCGA. With adjusted *p* value < 0.05 and |log2 fold-change|> 1, 687 up- and 566 down-regulated DEGs were determined in TNBC than normal tissues (Fig. 2A–C; Supplementary Table 1). The up-regulated DEGs were notably connected to immune responses and immunity, with the down-regulated DEGs being primarily linked with response to lipid, cell migration, and angiogenesis (Fig. 2D, E). In addition, KEGG pathways, e.g., protein processing in endoplasmic reticulum, antigen processing and presentation, proteasome, phagosome, cell adhesion molecules and oxidative phosphorylation were notably enriched by the up-regulated DEGs, with PPAR signaling pathway, regulation of lipolysis in adipocytes and adipocytokine signaling pathway being notably enriched by the down-regulated DEGs (Fig. 2F, G). The above data uncovered the crucial functions of the DEGs in TNBC progression. On the basis of the chemical formula from the PubChem database (CC1CCC2(CCC3(C(= CCC4C3(CCC5C4(CCC(C5(C)C)O)C)C)C2C1C)C)C(= O)O), 100 potential UA targets were predicted via the SwissTargetPrediction (Table 1). Following intersection with the DEGs, *FABP4*, *GLUL*, *MMP2*, *NR3C1*, *PPARG*, *PTPRF*, and *TOP2A* were determined as UA targets against TNBC (Fig. 2H), with close protein–protein interactions (Fig. 2I). Among them, *FABP4*, *GLUL*, *MMP2*, *NR3C1*, and *PPARG* presented down-regulation in TNBC, with *PTPRF* and *TOP2A* presenting up-regulation in TNBC (Fig. 2J).

**Table 1:** List of UA targets predicted by the SwissTargetPrediction

Target	Uniprot ID	ChEMBL ID	Target class	Probability	Known actives (3D/2D)
PTPN1	P18031	CHEMBL335	Phosphatase	0.95322338	46/72
POLB	P06746	CHEMBL2392	Enzyme	0.81269521	5/12
AKR1B10	O60218	CHEMBL5983	Enzyme	0.81269521	7/7
RORC	P51449	CHEMBL1741186	Nuclear receptor	0.72915815	1/10
PTPRF	P10586	CHEMBL3521	Membrane receptor	0.72915815	1/1
PTPN2	P17706	CHEMBL3807	Phosphatase	0.72915815	13/24
HSD11B1	P28845	CHEMBL4235	Enzyme	0.72915815	41/41
ACP1	P24666	CHEMBL4903	Phosphatase	0.72915815	1/2
PDE4D	Q08499	CHEMBL288	Phosphodiesterase	0.64536304	1/1
PLA2G1B	P04054	CHEMBL4426	Enzyme	0.58663687	1/1
CDC25B	P30305	CHEMBL4804	Phosphatase	0.58663687	4/6
CD81	P60033	CHEMBL1075180	Surface antigen	0.51122798	1/1
PTGES	O14684	CHEMBL5658	Enzyme	0.16766828	21/13
TERT	O14746	CHEMBL2916	Enzyme	0.11738988	1/2
FAAH	O00519	CHEMBL2243	Enzyme	0.10901347	0/13
FABP3	P05413	CHEMBL3344	Fatty acid binding protein family	0.10901347	0/5
FABP5	Q01469	CHEMBL3674	Fatty acid binding protein family	0.10901347	0/2
PPARD	Q03181	CHEMBL3979	Nuclear receptor	0.10901347	0/11
FABP1	P07148	CHEMBL5421	Fatty acid binding protein family	0.10901347	0/3
NOS2	P35228	CHEMBL4481	Enzyme	0.10901347	10/20
PTPN6	P29350	CHEMBL3166	Phosphatase	0.10901347	1/2
SCD	O00767	CHEMBL5555	Enzyme	0.10901347	0/1
CDC25A	P30304	CHEMBL3775	Phosphatase	0.10063443	3/16
PPARG	P37231	CHEMBL235	Nuclear receptor	0.10063443	46/24
PTPN11	Q06124	CHEMBL3864	Phosphatase	0.10063443	2/1
CYP19A1	P11511	CHEMBL1978	Cytochrome P450	0.10063443	0/258
FNTA FNTB	P49354 P49356	CHEMBL2094108	Enzyme	0.10063443	24/14
NR3C2	P08235	CHEMBL1994	Nuclear receptor	0.10063443	0/25
AR	P10275	CHEMBL1871	Nuclear receptor	0.10063443	1/128
SERPINA6	P08185	CHEMBL2421	Secreted protein	0.10063443	0/23
SHBG	P04278	CHEMBL3305	Secreted protein	0.10063443	0/54
G6PD	P11413	CHEMBL5347	Enzyme	0.10063443	0/7
CYP51A1	Q16850	CHEMBL3849	Cytochrome P450	0.10063443	0/2
PTGS2	P35354	CHEMBL230	Oxidoreductase	0.10063443	2/5
PREP	P48147	CHEMBL3202	Protease	0.10063443	1/2
PTGS1	P23219	CHEMBL221	Oxidoreductase	0.10063443	0/3
FFAR1	O14842	CHEMBL4422	Family A G protein-coupled receptor	0.10063443	0/3
HSD11B2	P80365	CHEMBL3746	Enzyme	0.10063443	4/19
PPARA	Q07869	CHEMBL239	Nuclear receptor	0.10063443	8/17
CES2	O00748	CHEMBL3180	Enzyme	0.10063443	1/14
NPC1L1	Q9UHC9	CHEMBL2027	Other membrane protein	0.10063443	0/11
SIGMAR1	Q99720	CHEMBL287	Membrane receptor	0.10063443	0/4
CYP17A1	P05093	CHEMBL3522	Cytochrome P450	0.10063443	0/43
ESR2	Q92731	CHEMBL242	Nuclear receptor	0.10063443	3/46
LTB4R	Q15722	CHEMBL3911	Family A G protein-coupled receptor	0.10063443	12/6
FABP4	P15090	CHEMBL2083	Fatty acid binding protein family	0.10063443	1/4
SAE1 UBA2	Q9UBE0 Q9UBT2	CHEMBL2095174	Enzyme	0.10063443	1/1
NR3C1	P04150	CHEMBL2034	Nuclear receptor	0.10063443	10/38
SLC6A3	Q01959	CHEMBL238	Electrochemical transporter	0.10063443	0/2
ADORA3	P0DMS8	CHEMBL256	Family A G protein-coupled receptor	0.10063443	0/3
SLC6A4	P31645	CHEMBL228	Electrochemical transporter	0.10063443	0/5
CYP2C19	P33261	CHEMBL3622	Cytochrome P450	0.10063443	0/4
TOP1	P11387	CHEMBL1781	Isomerase	0.10063443	0/2
PGR	P06401	CHEMBL208	Nuclear receptor	0.10063443	3/46
NR1H3	Q13133	CHEMBL2808	Nuclear receptor	0.10063443	1/20
PRKCH	P24723	CHEMBL3616	Kinase	0.10063443	2/1
PTGER2	P43116	CHEMBL1881	Family A G protein-coupled receptor	0.10063443	31/24
HMGCR	P04035	CHEMBL402	Oxidoreductase	0.10063443	20/68
CHRM2	P08172	CHEMBL211	Family A G protein-coupled receptor	0.10063443	0/1
SLC6A2	P23975	CHEMBL222	Electrochemical transporter	0.10063443	0/3
ALOX5	P09917	CHEMBL215	Oxidoreductase	0.10063443	12/21
GRIK1	P39086	CHEMBL1918	Ligand-gated ion channel	0.10063443	0/26
GRIK2	Q13002	CHEMBL3683	Ligand-gated ion channel	0.10063443	0/8
PTGER4	P35408	CHEMBL1836	Family A G protein-coupled receptor	0.10063443	56/9
HSF1	Q00613	CHEMBL5869	Other cytosolic protein	0.10063443	1/0
RORA	P35398	CHEMBL5868	Nuclear receptor	0.10063443	0/4
GPBAR1	Q8TDU6	CHEMBL5409	Family A G protein-coupled receptor	0.10063443	10/14
NR1H4	Q96RI1	CHEMBL2047	Nuclear receptor	0.10063443	7/8
NR1I3	Q14994	CHEMBL5503	Nuclear receptor	0.10063443	0/2
CES1	P23141	CHEMBL2265	Enzyme	0.10063443	0/4
BACE1	P56817	CHEMBL4822	Protease	0.10063443	5/2
MDM2	Q00987	CHEMBL5023	Other nuclear protein	0	24/0
TRPV1	Q8NER1	CHEMBL4794	Voltage-gated ion channel	0	0/2
POLA1	P09884	CHEMBL1828	Transferase	0	0/6
SLC22A6	Q4U2R8	CHEMBL1641347	Electrochemical transporter	0	0/2
BCHE	P06276	CHEMBL1914	Hydrolase	0	1/3
PTGIR	P43119	CHEMBL1995	Family A G protein-coupled receptor	0	2/8
TOP2A	P11388	CHEMBL1806	Isomerase	0	1/2
ESR1	P03372	CHEMBL206	Nuclear receptor	0	4/39
PTGDR2	Q9Y5Y4	CHEMBL5071	Family A G protein-coupled receptor	0	16/0
CNR1	P21554	CHEMBL218	Family A G protein-coupled receptor	0	2/7
AGTR1	P30556	CHEMBL227	Family A G protein-coupled receptor	0	55/0
ALOX5AP	P20292	CHEMBL4550	Other cytosolic protein	0	15/0
MAPK3	P27361	CHEMBL3385	Kinase	0	1/1
IL6	P05231	CHEMBL1795129	Secreted protein	0	0/1
GLUL	P15104	CHEMBL4612	Ligase	0	0/1
MMP3	P08254	CHEMBL283	Protease	0	13/0
MMP1	P03956	CHEMBL332	Protease	0	8/0
MMP2	P08253	CHEMBL333	Protease	0	17/0
IDO1	P14902	CHEMBL4685	Enzyme	0	0/1
PTGER1	P34995	CHEMBL1811	Family A G protein-coupled receptor	0	23/12
SRD5A2	P31213	CHEMBL1856	Oxidoreductase	0	1/21
ITGAL	P20701	CHEMBL1803	Adhesion	0	0/3
SREBF2	Q12772	CHEMBL1795166	Unclassified protein	0	0/1
ACHE	P22303	CHEMBL220	Hydrolase	0	1/4
AMPD2	Q01433	CHEMBL2997	Enzyme	0	10/0
CTSA	P10619	CHEMBL6115	Protease	0	4/0
EDNRA	P25101	CHEMBL252	Family A G protein-coupled receptor	0	39/0
ECE1	P42892	CHEMBL4791	Protease	0	11/0
SRD5A1	P18405	CHEMBL1787	Oxidoreductase	0	0/6

### UA treatment up-regulates FABP4 and PPARG in TNBC cells in a concentration-based fashion

**Fig. 3:**
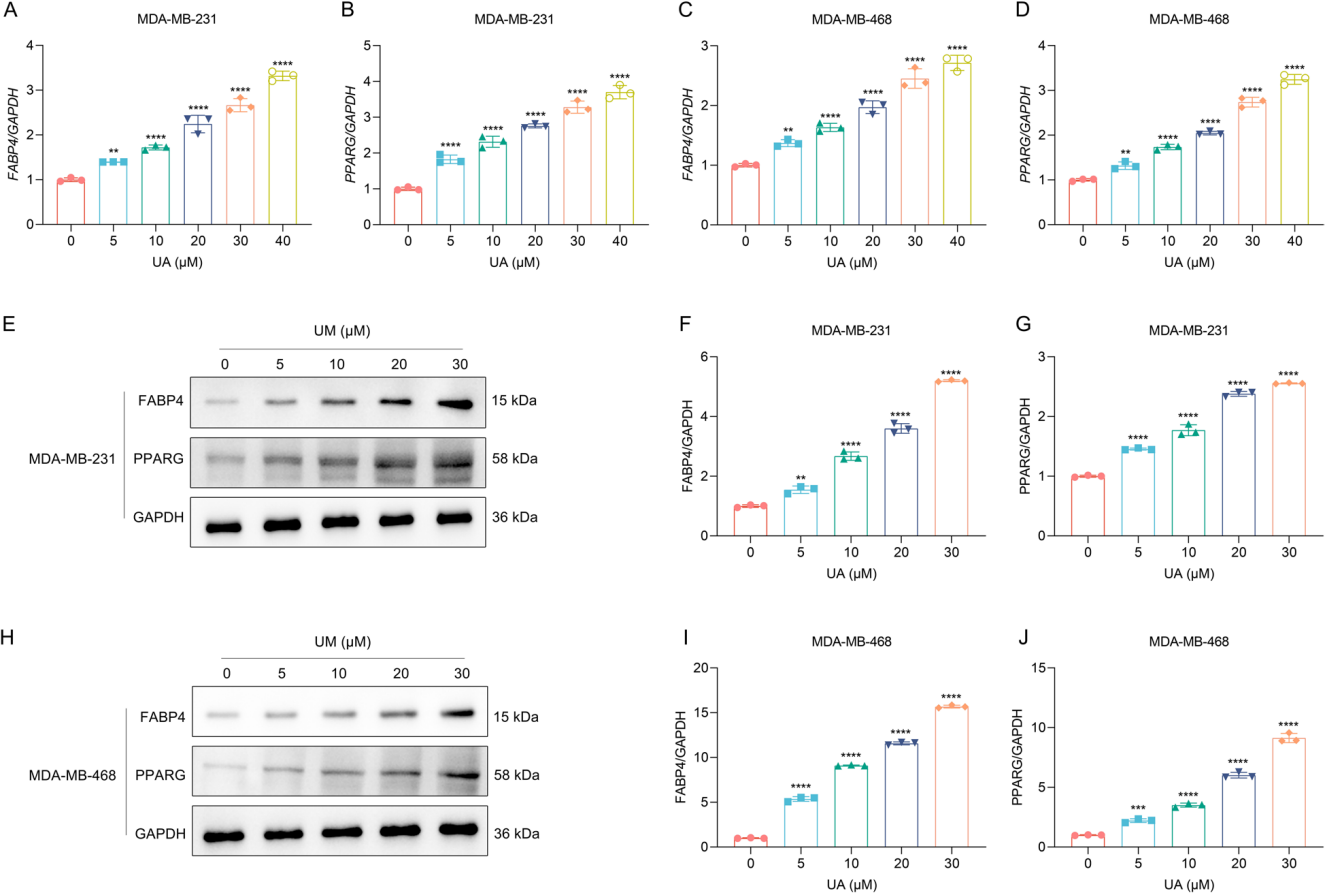
UA administration up-regulates FABP4 and PPARG expression in TNBC cells in a concentration-dependent fashion. **A**, **B** Detection of *FABP4* and *PPARG* mRNA levels in MDA-MB-231 cells with 48-h exposure to 0, 5, 10, 20, 30, and 40 μM UA that was dissolved by DMSO for 24, 48, and 72 h. DMSO was used as a vehicle control. **C**, **D** Detection of *FABP4* and *PPARG* mRNA levels in MDA-MB-468 cells with 48-h exposure to 0, 5, 10, 20, 30, and 40 μM UA. **E** Representative immunoblotting images of FABP4 and PPARG in MDA-MB-231 cells with 48-h exposure to 0, 5, 10, 20, and 30 μM UA. GAPDH acted as a control. **F**, **G** Quantitative analysis of FABP4 and PPARG levels in MDA-MB-231 cells with 48-h exposure to 0, 5, 10, 20, and 30 μM UA. **H** Representative immunoblotting images of FABP4 and PPARG in MDA-MB-468 cells administrated with 0, 5, 10, 20, and 30 μM UA for 48 h. GAPDH acted as a control. **I**, **J** Quantitative analysis of FABP4 and PPARG levels in MDA-MB-468 cells administrated with 0, 5, 10, 20, and 30 μM UA for 48 h. *n* = 3 per group. **, *p* < 0.01; ***, *p* < 0.001; ****, *p* < 0.0001 from one-way analysis of variance with Tukey post-test

Among the UA targets, *FABP4* and *PPARG* mRNA levels were verified to be gradually elevated in MDA-MB-231 and MDA-MB-468 cells exposed to a graded concentration of UA for 48 h (Fig. 3A–D). In addition, as the concentration of UA increased, FABP4 and PPARG protein levels presented gradual elevation in TNBC cells (Fig. 3E–J). Consequently, UA administration can up-elevate FABP4 and PPARG expression in TNBC cells in a concentration-based fashion, uncovering that FABP4 and PPARG may be therapeutic targets of UA in treating TNBC.

### FABP4 and PPARG are remarkably down-regulated in human TNBC tissues than normal tissues

**Fig. 4:**
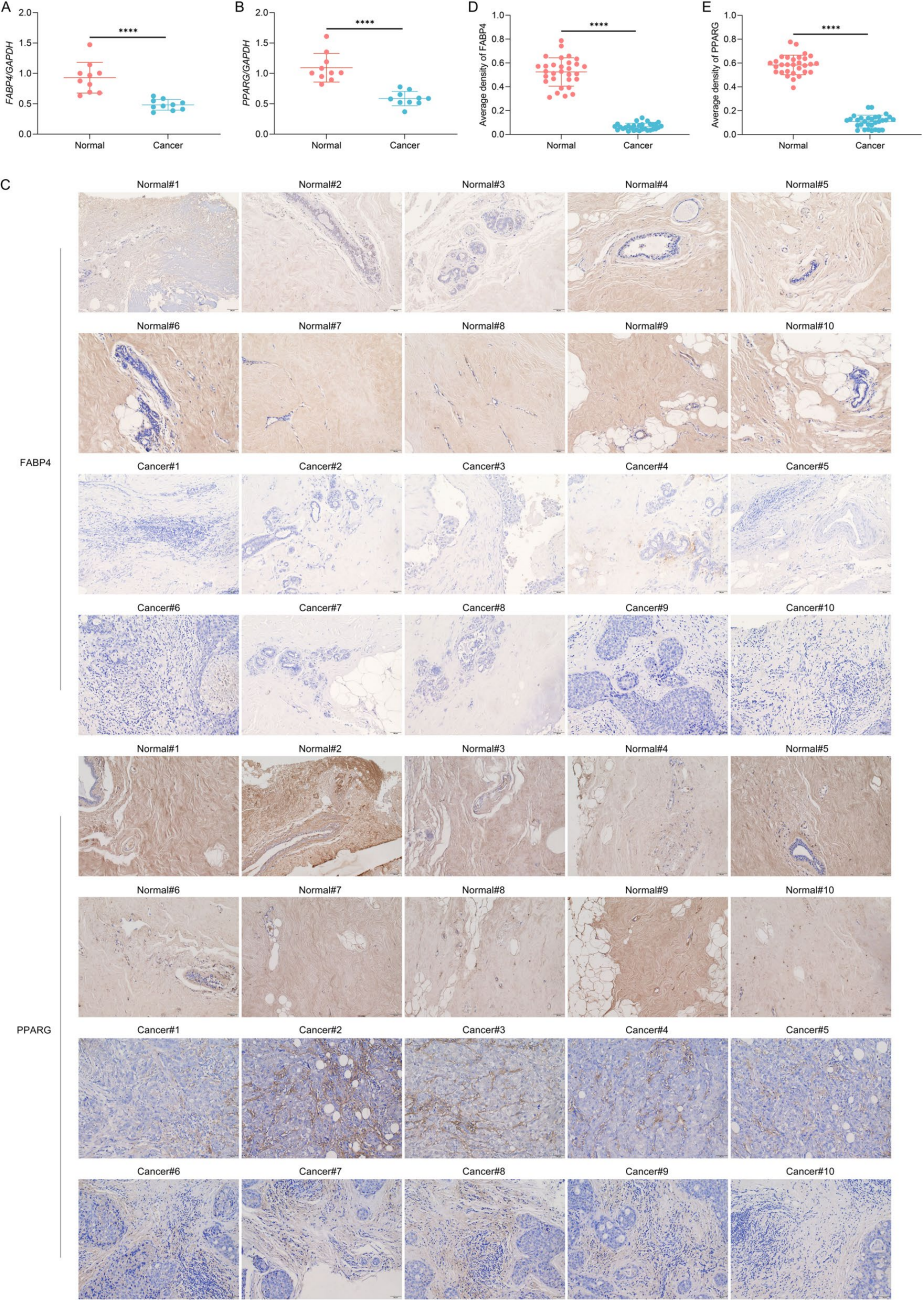
FABP4 and PPARG present notable down-regulation in human TNBC tissues versus normal tissues. **A**, **B** Detection of *FABP4* and *PPARG* mRNA levels in 10 pairs of human TNBC tumor tissues and matched normal tissues. **C** Representative immunohistochemical photographs of FABP4 and PPARG in 10 pairs of human TNBC tumor tissues and matched normal tissues. Scale bar, 50 μm. **D**, **E** Quantitative analysis of FABP4 and PPARG levels in 10 pairs of human TNBC tumor tissues and matched normal tissues. *n* = 10 per group. ****, *p* < 0.0001 from unpaired *t* test

To characterize the clinical relevance of FABP4 and PPARG, this study gathered 10 pairs of human TNBC tumor tissues and corresponding normal tissues. *FABP4* and *PPARG* mRNA levels were observed to be remarkably decreased in human TNBC tissues in comparison with normal tissues (Fig. 4A, B). Immunohistochemical staining demonstrated that FABP4 and PPARG were widely distributed in human normal tissues but lowly expressed in human TNBC tissues (Fig. 4C–E). The above findings uncover that the low expression of FABP4 and PPARG may be linked with TNBC occurrence and progression.

### FABP4 knockdown attenuates UA treatment-induced activation of FABP4/PPARG pathway in TNBC cells

**Fig. 5:**
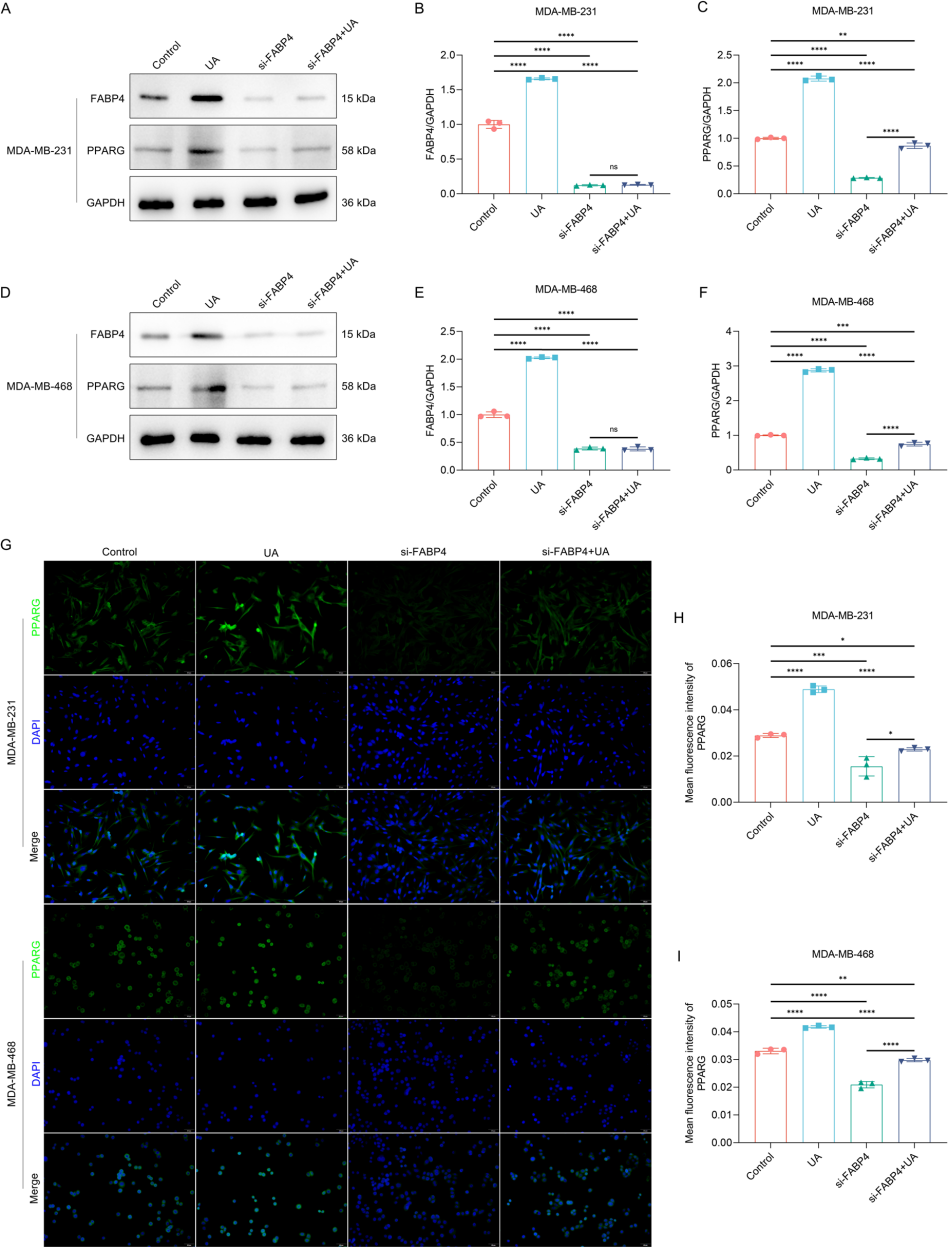
FABP4 knockdown prevents UA treatment-induced activation of FABP4/PPARG pathway in TNBC cells. **A** Representative immunoblotting images of FABP4 and PPARG in MDA-MB-231 cells transfected with si-FABP4 or/and administrated with UA for 48 h. DMSO and si-NC were used as a control of UA and si-FABP4, respectively. **B**, **C** Quantitative analysis of FABP4 and PPARG levels in MDA-MB-231 cells transfected with si-FABP4 or/and administrated with UA for 48 h. **D** Representative immunoblotting of FABP4 and PPARG in MDA-MB-468 cells with si-FABP4 transfection or/and UA administration for 48 h. **E**, **F** Quantitative analysis of FABP4 and PPARG levels in MDA-MB-468 cells with si-FABP4 transfection or/and UA administration for 48 h. **G** Representative immunofluorescent photographs of MDA-MB-231 and MDA-MB-468 cells transfected with si-FABP4 or/and administrated with UA for 48 h. Scale bar, 50 μm. **H**, **I** Quantitative analysis of PPARG expression in MDA-MB-231 and MDA-MB-468 cells with si-FABP4 transfection or/and UA administration for 48 h. *n* = 3 per group. *, *p* < 0.05; **, *p* < 0.01; ***, *p* < 0.001; ****, *p* < 0.0001 from one-way analysis of variance with Tukey post-test

To further verify the influence of UA treatment on FABP4/PPARG pathway, this study transfected si-FABP4/si-NC into MDA-MB-231 and MDA-MB-468 cells to silence FABP4 and subsequently administrated with UA/DMSO for 48 h. Among the three siRNAs of FABP4, si-FABP4#1 was found to have the best knockdown effect (Supplementary Fig. 2A–C), which was used for subsequent analysis. UA administration remarkably elevated FABP4 and PPARG expression, and such effects were remarkably reversed by si-FABP4 (Fig. 5A–F). Further immunofluorescence demonstrated that UA administration increased PPARG expression and heightened its activation, which were notably reversed by si-FABP4 (Fig. 5G–I). Altogether, FABP4 knockdown can reverse UA treatment-induced activation of FABP4/PPARG pathway in TNBC cells.

### FABP4 knockdown weakens the efficacy of UA treatment on suppression of growth and migration of TNBC cells

**Fig. 6:**
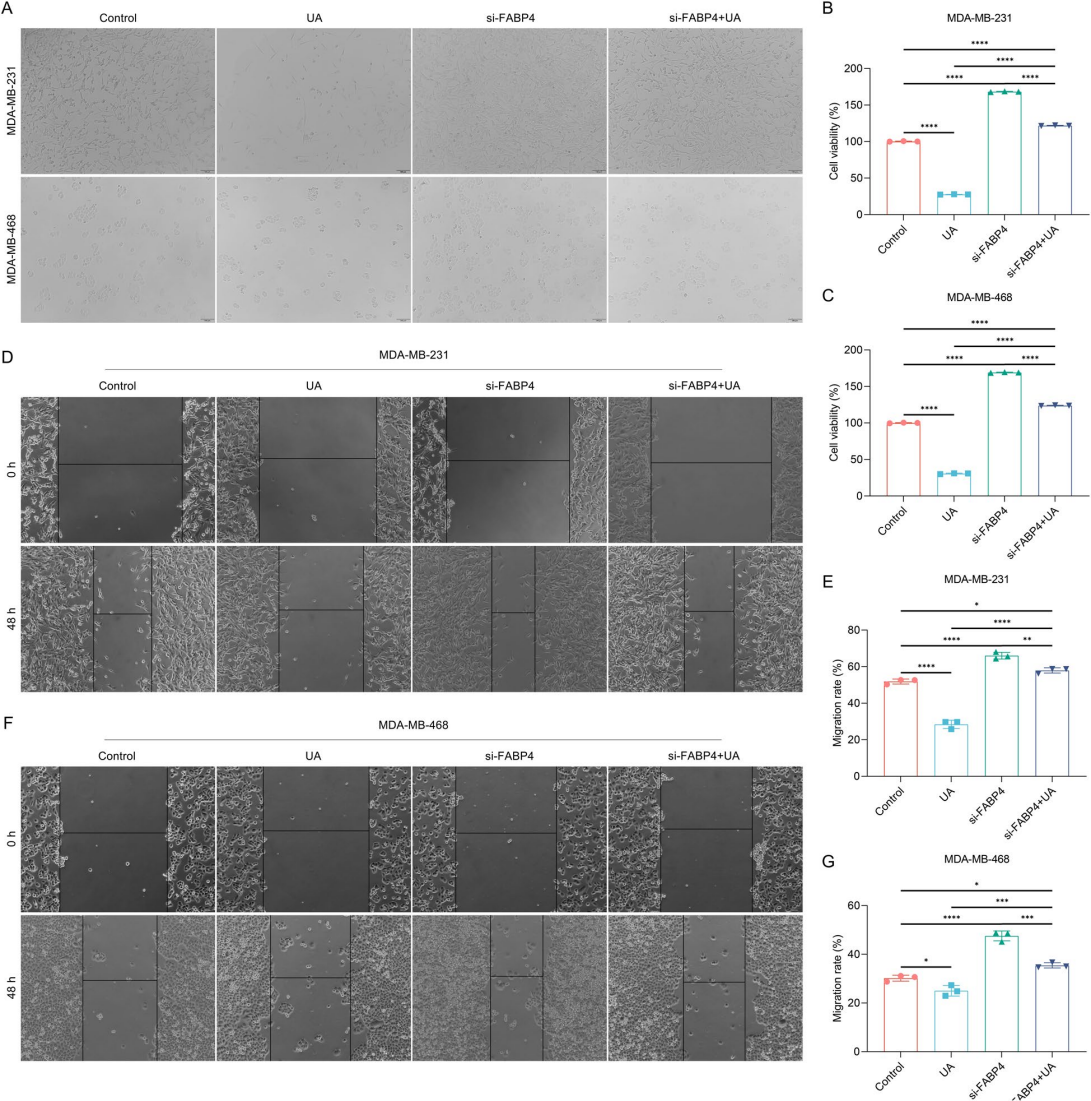
FABP4 knockdown reverses the effects of UA treatment on suppression of growth and migration of TNBC cells. **A** Photographs of MDA-MB-231 and MDA-MB-468 cells with si-FABP4 transfection or/and UA administration for 48 h. DMSO and si-NC were used as a control of UA and si-FABP4, respectively. Scale bar, 100 μm. **B**, **C** Cell proliferation of MDA-MB-231 and MDA-MB-468 cells with si-FABP4 transfection or/and UA administration for 48 h. **D** Photographs of MDA-MB-231 cells with si-FABP4 transfection or/and UA administration after scratching at 0 h, and 48 h. Scale bar, 100 μm. **E** Calculation of migration rate of MDA-MB-231 cells with si-FABP4 transfection or/and UA administration. **F** Photographs of MDA-MB-468 cells with si-FABP4 transfection or/and UA administration after scratching at 0 h, and 48 h. Scale bar, 100 μm. **G** Calculation of migration rate of MDA-MB-468 cells with si-FABP4 transfection or/and UA administration. *n* = 3 per group. *, *p* < 0.05; **, *p* < 0.01; ***, *p* < 0.001; ****, *p* < 0.0001 from one-way analysis of variance with Tukey post-test

UA administration had an effect on inhibiting MDA-MB-231 and MDA-MB-468 cell proliferation, and such effect was prominently attenuated when FABP4 expression was silenced (Fig. 6A–C). In addition, the suppression of migratory potential of TNBC cells by UA administration was remarkably weakened by FABP4 knockdown (Fig. 6D–G). Collectively, silencing FABP4 can reverse the efficacy of UA treatment on suppression of TNBC cell growth and migration.

### Overexpression of FABP4/PPARG enhances the inhibitory effect of UA on growth and migration of TNBC cells

**Fig. 7:**
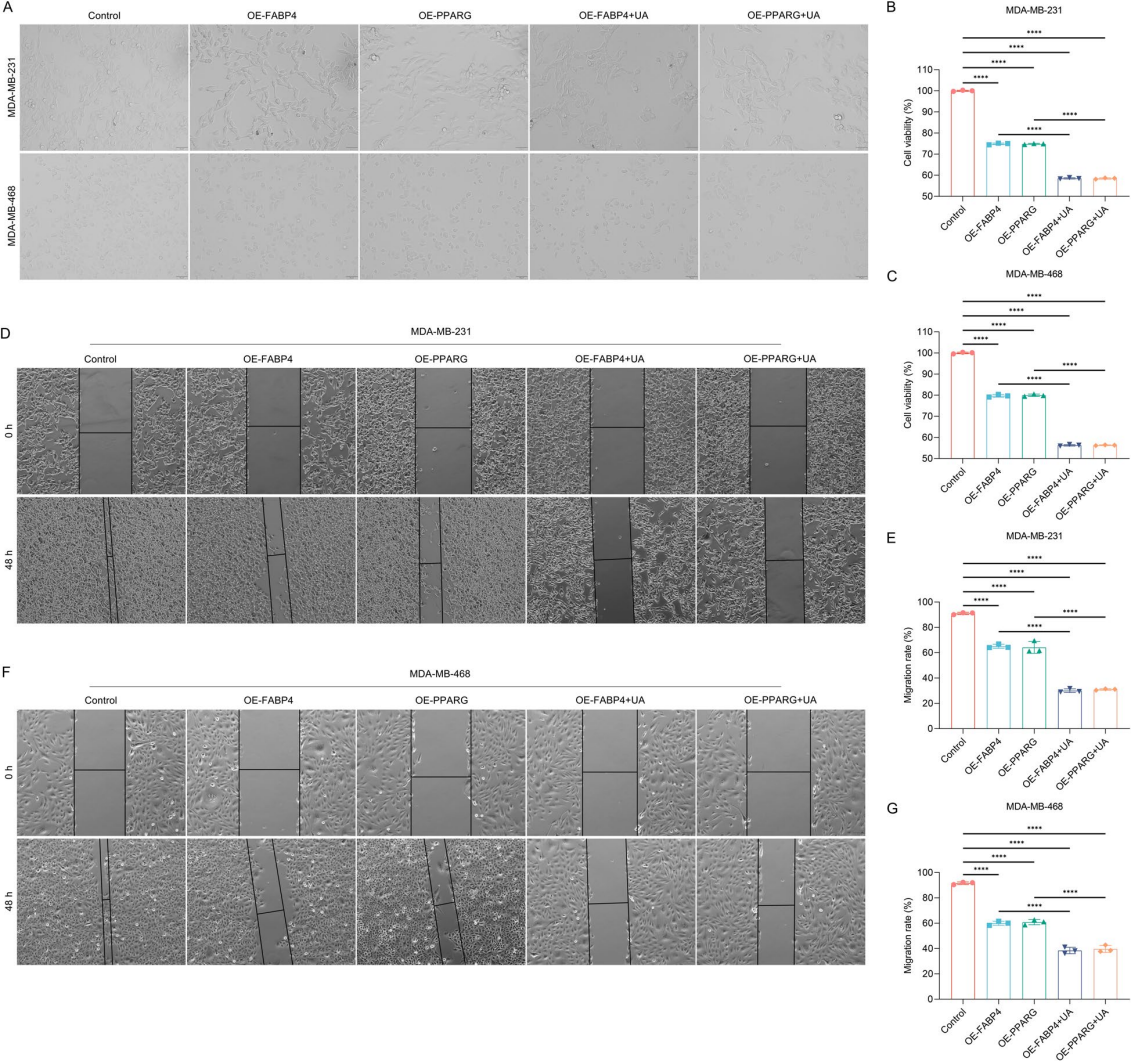
Overexpression of FABP4/PPARG enhances the inhibitory effect of UA on growth and migration of TNBC cells. **A** Photographs of MDA-MB-231 and MDA-MB-468 cells with FABP4/PPARG overexpression or/and UA administration for 48 h. DMSO and empty vector were used as a control of UA and FABP4/PPARG overexpression, respectively. Scale bar, 50 μm. **B**, **C** Cell proliferation of MDA-MB-231 and MDA-MB-468 cells with FABP4/PPARG overexpression or/and UA administration for 48 h. **D** Photographs of MDA-MB-231 cells with FABP4/PPARG overexpression or/and UA administration after scratching at 0 h, and 48 h. Scale bar, 100 μm. **E** Calculation of migration rate of MDA-MB-231 cells with FABP4/PPARG overexpression or/and UA administration. **F** Photographs of MDA-MB-468 cells with FABP4/PPARG overexpression or/and UA administration after scratching at 0 h, and 48 h. Scale bar, 100 μm. **G** Calculation of migration rate of MDA-MB-468 cells with FABP4/PPARG overexpression or/and UA administration. *n* = 3 per group. ****, *p* < 0.0001 from one-way analysis of variance with Tukey post-test

To further validate that FABP4/PPARG pathway is a therapeutic target for UA, we overexpressed FABP4/PPARG in MDA-MB-231 and MDA-MB-468 cells, in combination with UA treatment. As a result, the combination of overexpressed FABP4/PPARG with UA synergistically inhibited proliferation (Fig. 7A–C) and migration (Fig. 7D–G) of TNBC cells. Taken together, overexpression of the FABP4/PPARG pathway boosts the inhibitory effect of UA on growth and migration of TNBC cells.

## Discussion

UA is a naturally synthesized pentacyclic triterpenoid, which possesses a wide range of pharmacological activities and modulates several crucial signaling pathways, such as STATs, NF-κB, and TRAIL [33, 34]. The present study evaluated the antitumor efficacy of UA against TNBC as well as the underlying mechanisms, and uncovered that UA treatment enabled to prevent TNBC progression via mediating FABP4/PPARG pathway.

Our data demonstrated that UA treatment lowered growth and migration of TNBC cells in a concentration-based fashion, which was similar to prior studies. For instance, it was reported that UA can attenuate proliferation of TNBC stem‑like cells via activating NRF2‑mediated ferroptosis [19]. UA can also prevent TNBC cell proliferation through decreasing PLK1 and CCNB1 expression via modulation of p53 pathway [35]. Furthermore, UA has been demonstrated to heighten response of TNBC cells to doxorubicin based on ZEB1-AS1/miR-186-5p/ABCC1 axis [36]. In TNBC cells, RORγ acted as a direct target of UA, which reversed RORγ-mediated cellular proliferation, apoptosis and cholesterol biosynthesis [37]. In addition, UA–liposomes treatment can also modulate tumor microenvironment of TNBC and reduce immunosuppressive cells (e.g., MDSCs and Tregs), with a potential as an immunotherapy agent [38]. Combining with prior findings, we believe that UA is a potent natural compound for treating TNBC.

To clarify the underlying mechanisms of UA, we determined DEGs between TNBC and normal tissues, and predicted possible UA targets by use of the SwissTargetPrediction. Consequently, *FABP4*, *GLUL*, *MMP2*, *NR3C1*, *PPARG*, *PTPRF*, and *TOP2A* were discovered as UA targets against TNBC, of which *FABP4*, *GLUL*, *MMP2*, *NR3C1*, and *PPARG* showed down-regulation in TNBC as well as *PTPRF* and *TOP2A* presented up-regulation in TNBC. After experimental verification, FABP4 and PPARG mRNA and protein levels were elevated by UA administration in a concentration-based manner. We inferred that FABP4 and PPARG were direct targets of UA. Clinically, FABP4 and PPARG levels presented notable reduction in TNBC versus normal tissues. It was reported that hypoxic condition can elevate the expression of FABP4 and PPARG in TNBC cells [39]. FABP4-mediated lipid metabolism facilitated TNBC progression and cancer stem cell characteristics [6]. CD36^+^ fibroblasts secreted SLIT3, FBLN-1, and PENK three ligands to inhibit TNBC growth and to elevate FABP4 expression in TNBC cells [40]. In addition to UA, asiaticoside can also hamper TNBC epithelial–mesenchymal transition through elevating PPARG levels and inhibiting P2RX7-based TGF-β/Smad pathway [15]. VSP‑17 enables to prevent migration and invasion of TNBC cells via hampering EMT process by PPARG/AMPK pathway [41]. MicroRNA-27b-3p heightens TNBC progression and metastases through suppression of PPARG pathway [42]. Epigenetic de-repression transforms PPARG into a drug target for TNBC and endocrine-resistant breast cancer [43]. Altogether, we inferred that UA treatment may transcriptionally activate and epigenetically modulate FABP4 and PPARG, thus preventing TNBC cell proliferation and migration.

There is a positive feedback loop between FABP4 and PPARG in promoting fatty acid uptake and oxidation in lymph node metastasis of cholangiocarcinoma [13]. Herein, we observed that FABP4 knockdown effectively reversed the elevation of FABP4 and PPARG expression induced by UA administration in TNBC cells. Especially, UA up-regulated PPARG expression and heightened its activation, which were also reversed by FABP4 knockdown. In addition, FABP4 knockdown weakened the efficacy of UA administration on suppression of TNBC cell growth and migration, thus preventing UA-induced TNBC progression suppression, revealing that UA suppresses TNBC progression partly through activation of FABP4/PPARG pathway. More experiments will be required for confirming our findings.

The limitations of this study should be pointed out. Considering lower clinically relevant concentrations (0.1 to 2 μM), the clinical therapeutic value of UA needs to be further evaluated, since we used the high UA doses. UA can be classified as a class IV drug in biopharmaceutics classification system, thus limiting its development as an oral drug. It is important to increase the bioavailability of UA through improved technical processes, such as using nanocarriers and nanoparticles. Furthermore, toxicity studies need to be conducted to assess adverse effects of UA.

## Conclusion

Collectively, our findings uncover that UA possess a potent effect on preventing TNBC cell proliferation and migration through activating FABP4/PPARG pathway. Nevertheless, the antitumor effects of UA against TNBC and the involved mechanisms should be verified in vivo experiments in our future studies.

## Supplementary Information

Additional file 1.

Additional file 2: Supplementary table 1. The list of DEGs in TNBC versus normal tissues.

Additional file 3: Supplementary figure 1. Influence of FABP4 knockdown on proliferation of normal breast epithelial cells. (A) Photographs of MCF-10A cells with exposure to 0, 5, 10, 20, and 30 μM UA that was dissolved by DMSO for 24, 48, and 72 h. DMSO was used as a vehicle control. Scale bar, 50 μm. (B) Cell proliferation of MCF-10A cells with exposure to 0, 5, 10, 20, and 30 μM UA for 24, 48, and 72 h. n=3 per group. ****, p < 0.0001 from one-way analysis of variance with Tukey post-test.

Additional file 4: Supplementary figure 2. Knockdown effect of FABP4 siRNAs in TNBC cells. (A) Representative immunoblotting images of FABP4 in MDA-MB-231 and MDA-MB-468 cells transfected with si-FABP4/si-NC. (B, C) Quantitative analysis of FABP4 expression. n=3 per group. ****, p < 0.0001 from one-way analysis of variance with Tukey post-test.

## Data Availability

No datasets were generated or analysed during the current study.

## Abbreviations

TNBC: Triple-negative breast cancer
FABPs: Fatty acid binding proteins
PPARG: Peroxisome proliferator-activated receptor gamma
UA: Ursolic acid
DEGs: Differentially expressed genes
GO: Gene Ontology
KEGG: Kyoto Encyclopedia of Genes and Genomes
