# Supplementary material for: Ursolic acid suppresses triple-negative breast cancer progression through mediating FABP4/PPARG pathway
Source: Eur J Med Res. 2025 Jul 2;30:550. doi: 10.1186/s40001-025-02794-y (PMC12219972; doi:10.1186/s40001-025-02794-y)

Figure 3E | Figure 3H | Figure 5A | Figure 5D

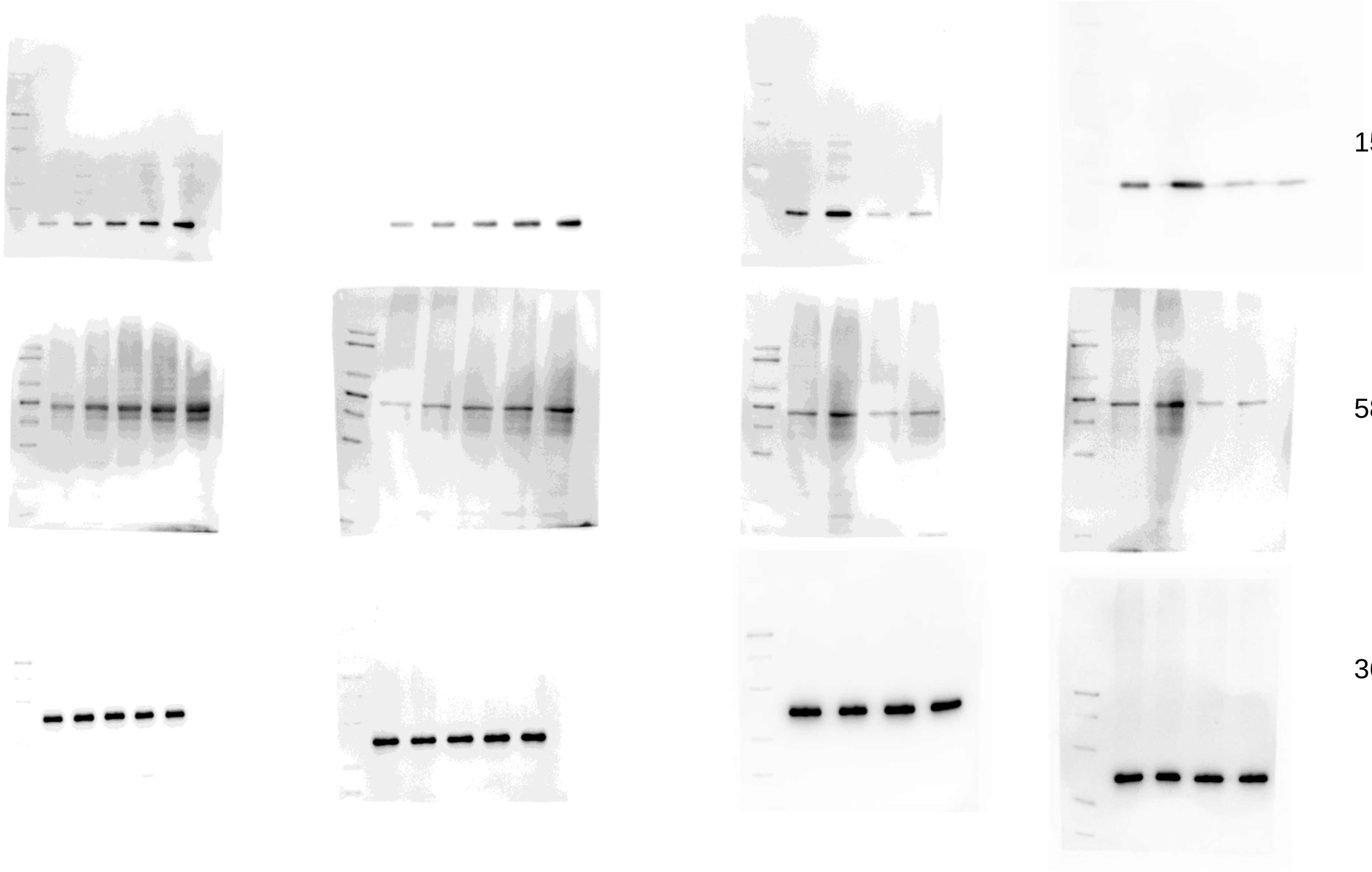

Supplementary figure 2A

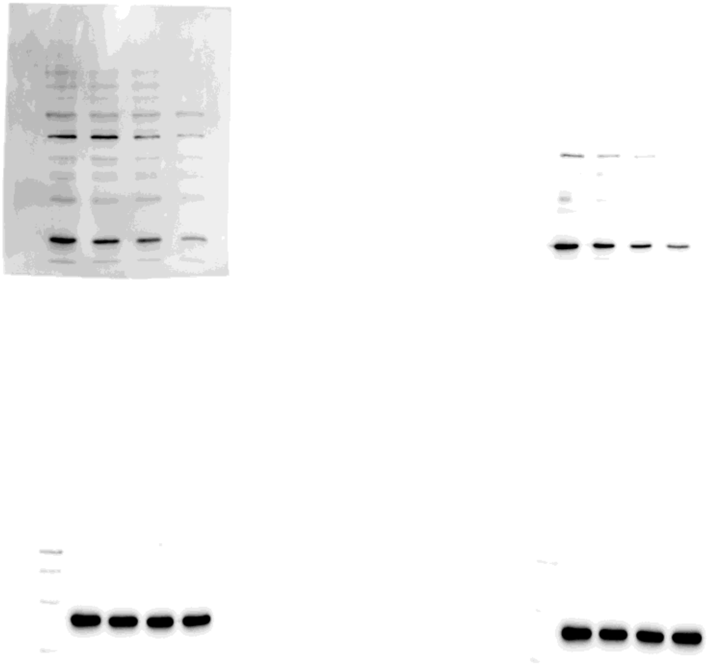

Supplement: Supplementary file 1 — Additional file 1. [file 40001_2025_2794_MOESM1_ESM.pdf]
