# Supplementary figures and images for: Ursolic acid suppresses triple-negative breast cancer progression through mediating FABP4/PPARG pathway
Source: Eur J Med Res. 2025 Jul 2;30:550. doi: 10.1186/s40001-025-02794-y (PMC12219972; doi:10.1186/s40001-025-02794-y)

A

MCF-10A

UM ( $\mu\text{M}$ )

0

5

10

20

30

24 h

48 h

72 h

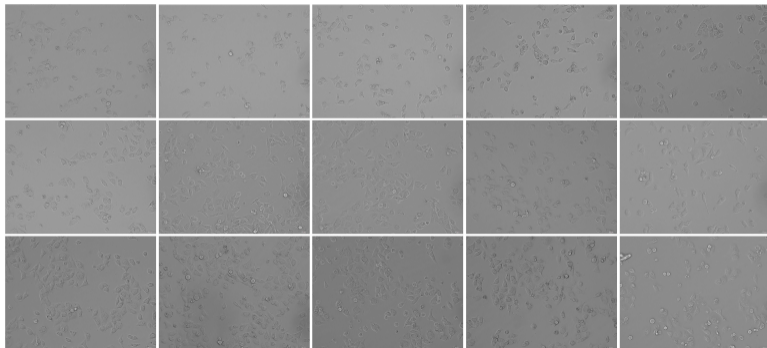

B

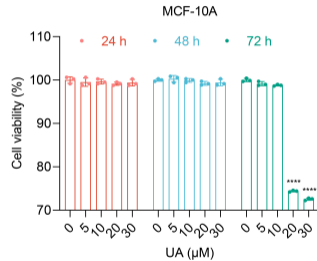

Supplement: Supplementary file 2 — Additional file 2: Supplementary table 1. The list of DEGs in TNBC versus normal tissues. [file 40001_2025_2794_MOESM2_ESM.pdf]

A

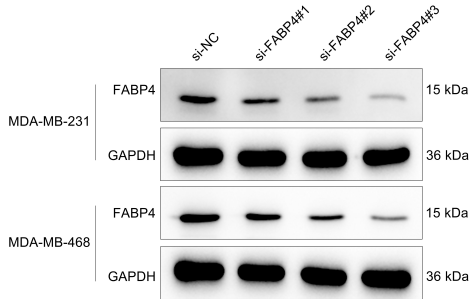

B

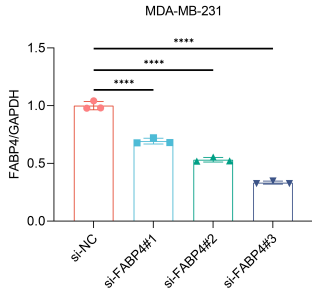

C

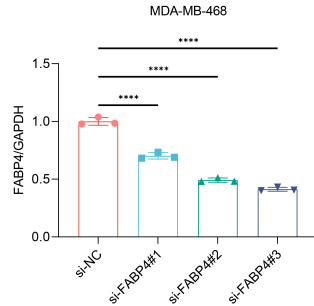

Supplement: Supplementary file 3 — Additional file 3: Supplementary figure 1. Influence of FABP4 knockdown on proliferation of normal breast epithelial cells. (A) Photographs of MCF-10A cells with exposure to 0, 5, 10, 20, and 30 μM UA that was dissolved by DMSO for 24, 48, and 72 h. DMSO was used as a vehicle control. Scale bar, 50 μm. (B) Cell proliferation of MCF-10A cells with exposure to 0, 5, 10, 20, and 30 μM UA for 24, 48, and 72 h. n=3 per group. ****, p < 0.0001 from one-way analysis of variance with Tukey post-test. [file 40001_2025_2794_MOESM3_ESM.pdf]
